# Specific antenatal interventions for Black, Asian and Minority Ethnic (BAME) pregnant women at high risk of poor birth outcomes in the United Kingdom: a scoping review

**DOI:** 10.1186/s12884-015-0657-2

**Published:** 2015-09-24

**Authors:** Rebecca Garcia, Nasreen Ali, Chris Papadopoulos, Gurch Randhawa

**Affiliations:** The Institute For Health Research, University of Bedfordshire, Putteridge Bury, Hitchin Road, Luton, Bedfordshire LU2 8LE UK

## Abstract

**Background:**

Disparity exists in maternal and infant birth outcomes of Black and Minority Ethnic (BAME) women giving birth in the United Kingdom (UK) compared to the majority. There is therefore a need to reconsider existing maternity service provision to ensure culturally competent services. The purpose of this scoping review was to ascertain what specific maternity interventions have been implemented in the UK for BAME women (2004–2014) so that increased awareness of the need and scope of specific maternity interventions for BAME women can be identified.

**Methods:**

A scoping review was conducted in order to determine the evidence base. It was determined that no prior systematic reviews had been conducted and it was apparent that literature in this field was sparse. Scoping review is an ideal method when literature is likely to be heterogeneous and the research field relatively unexplored. A keyword strategy was used implementing population (P), intervention (I), comparison (C) and outcomes (O).

**Results:**

An initial 2188 papers were identified. Following screening and review, only 5 heterogeneous papers remained suitable and were included. The included interventions employed sample sizes of *N* = 160-1441, examined a range of different outcome measures and were delivered across different parts of the UK with high numbers of BAME residents.

**Conclusions:**

There is a lack of rigorous research interventions and practice interventions which are currently documented, of specific maternity interventions which are aimed to address culturally competent maternity services and the sharing of best practice addressing the increased risks of BAME women delivering in the UK.

## Background

Adverse pregnancy outcomes such as maternal death during or soon after delivery, intrapartum stillbirth (defined as an infant born with no life signs after 24 weeks gestation) [1]; low birth weight (LBW) (defined as birth weight <2500 g) [2] and infant mortality (death of infant <1 year old) have steadily declined across the world [3], in response to advancing medical interventions, improved sanitation and better nutrition in recent decades [4]. However, maternal and infant health inequalities between and within developed and developing countries continue to persist [5, 6].

In the U.K., there are disparities in maternal mortality rates; the estimated white British maternal death rate is 8 per 100,000 maternities, compared to 28.05 for the Black ethnic^1^ group (combined); 32.82 for Black Africans, 31.89 for Black Caribbeans, 12.24 for Asians (Indian, Pakistani and Bangladeshi’s), 12.52 for Pakistani, and 12.47 for Bangladeshi [7, 8]. There are similar discrepant trends evident in the statistics of infant birth outcomes including stillbirth, pre-term delivery and perinatal mortality from BAME women in UK [9–11].

Low birth weight is an established risk factor for infant mortality [12, 13]. Infant mortality rate for babies in the UK whose weight is less than 1500 g (at birth) is 173 per 1000 live births whereas the rate for babies whose weight is less than or in excess of 2500 g at birth is 35.2 and 1.3 per 1000 live births respectively [1]. Research has also shown that low birth weight is commonly experienced in infants of South Asian mothers in the UK, with babies on average being 280–350 g lighter than white British infants [14, 15] and is an issue that has remained pervasive within the South Asian community for several generations [16–20]. Moreover, on average Black Caribbean infants are reported to be 150 g lighter than white British infants with a 60 % increase in the chance that Black African infants are more likely to be low birth weight, compared with white British infants [14].

The suggested explanations seen between the difference in prevalence is complex and multifaceted; involving physiological factors (e.g. small maternal stature, obesity, maternal age at conception and co-morbidity), deprivation, maternal health behaviours (e.g. smoking [active or passive], late booking, breastfeeding and social and cultural influences (e.g. spacing between pregnancies, levels of social support) [8, 21, 22]. A number of risk factors have been presented as contributing to adverse outcomes of maternal mortality, still births, low birth weight and infant mortality in the UK; some of these include suboptimal levels of care (Confidential Enquiry into Maternal Deaths, 2011), late booking (defined as booking after 13 weeks and 6 days) [23, 24]; delays in help seeking behaviours, delays in organisational procedures in prompt referral and management of pregnancy risks and/or complications [8, 25], and intrauterine growth restriction [26].

There is a wealth of research in developed countries to demonstrate BAME women experience barriers in accessing antenatal services. These include language barriers; whereby women do not have access to properly skilled translators (this includes health literate translators) [27–29]; unawareness of service provision or how to access services [30]; poor access to female health care staff, embarrassment of unknown male medical staff [31, 32]; physical restrictions due to socio-demographic limitations such as hospital proximity and access to transport [33, 34] and poor previous experience of health care services or stereotyped expectations from health care staff resulting in reductions in information giving and informed choices [32, 35]. Other contributory risk factors, such as socioeconomic status, including education status and income, and living in areas of high deprivation are frequently cited as distal determinants of poorer health outcomes [14, 22, 36].

Another commonly cited barrier to the utilisation of maternity service is lack of financial resource (or insurance) in order to pay for health or maternity services, however this does not apply in the U.K. context due to the wide availability of ‘*free’* antenatal or maternity services available to lawful residents [24, 37, 38]. However, despite having such access to healthcare and maternity services which are ‘*free at the point of delivery*’ in the U.K., two ethnic communities have been identified at pervasive and higher risk of adverse birth outcomes in the U.K., namely; Black Caribbean/Africans and South Asians [38–41].

Therefore, it is clear that multifaceted factors contribute to the continued inequalities evidenced in adverse maternal and infant outcomes seen in BAME infants born in the U.K.^2^ [42]; Despite them having access to ‘*free*’ maternity services in the NHS. Such inequality will undoubtedly contribute to the disproportionate adverse outcomes evidenced in BAME pregnant women. Moreover, evidence suggests that there are a number of the contributory factors for adversity for maternal and poor infant outcomes are considered to be modifiable (e.g. smoking, obesity, consanguinity) [43-44], therefore it is clear that, maternity services have a significant role to play in early identification and reduction of risk for women at disproportionate probability of adverse outcomes during their maternity experience [45, 46].

Maternity healthcare providers need to modify their current services to include culturally competent service provision, meeting the diverse needs of the evolving demographic profiles of the U.K. (as with other similar developed countries, e.g. European countries, Canada, USA and Australia). However, what remains unclear are which specific maternity interventions are currently being provided for high risk ethnic groups such as Black Caribbean or South Asians in United Kingdom in order to address the increased risks of adverse outcomes. Synthesising research evidence on current specific maternity interventions for BAME women in the UK will enable policy makers to modify services and develop services which can reduce inequalities and improve maternal and birth outcomes.

## Methods

### Study design

This review uses scoping methods. Methodological procedures for scoping review are currently not clearly defined, however this scoping review will follow the principals of Arksey and O’Malley’s framework [47], namely; identification of the research question, detection and sourcing of papers, study inclusion selection, charting of the data by either narrative terms or analytical terms and summarising results. The criteria for implementing scoping studies includes when the research question needs developing in an iterative way; the likely included studies use heterogeneous methodologies and scoping methods are also used to identify the extent and scope of current literature in the field, where the evidence base is somewhat limited. Following an initial search of the literature, it became evident that there was a paucity of population based random controlled trails exists to inform practice in this area. The methodologies of the final included studies were therefore anticipated to be heterogeneous, and it was anticipated that the final output of the review would be small, therefore satisfying the criteria for using scoping methods [47–50].

### Identifying the research question

Initially, the researchers intended to conduct a systematic review methodology assessing specific BAME maternity interventions; however it became clear early on that there was very little evidence base on this from the UK context. The available evidence from the global context was heterogenetic, making meaningful comparison between the interventions difficult. Consequently, the research question was developed in an iterative way, following preliminary searches of the national and global literature, whereby it became evident that a scoping exercise was necessary to map the current evidence base in the UK [43]. Consequently the research question became; ‘What specific BAME maternity interventions exist for UK-based BAME women?’

### Searching strategy and study identification procedure

As can be seen in Table 1, the keyword strategy was based on population, intervention comparison, and outcomes (PICO) and used Boolean operators to combine search terms associated with the population of interest (i.e. BAME groups), pregnancy, intervention, outcomes and geographical region.

**Table 1:** Search terms

Population^a^	Pregnancy	Intervention	Outcome	Region
*Search operator*	*AND*	*AND*	*AND*	*AND*
Asian	Pregnan^a^	Intervention	Low birth weight	United Kingdom
India^a^	Antenatal	Evaluation	Mortality	U.K.
Pakistan^a^	Prenatal	Trial^a^	Morbidity	Britain
Bangladeshi	Postnatal	Programme	Infant Mortality	England
Africa^a^	Postpartum	Experiment^a^	“Still birth”	Wales
Black	Expectant	Assessment	“inter uterine growth”	“Northern Ireland”
Black and minority ethnic	gestat^a^		Fatality	
Traveller	gravid^a^		stillbirth	
Gypsy	Post-partum		still-birth	
BME	expecting		“inter uterine restriction”	
BAME			“inter uterine redardation”	
“minority ethnic”			“inter-uterine growth”	
Chinese			“inter-uterine restriction”	
Mediterranean				

^a^ *population* classified according to the Office National Statistics Ethnicity classification [51]

The databases PubMed/Medline, PubMed Central, Europe PubMed Medline, Medline with Full Text; Academic Search Elite SocINDEX with Full Text and E-Journals (EBSCO-Host), CINAHL, British Nursing Index, PSYCHINFO, PsychARTICLES, AMED, ASSIA, AMED, British Nursing Index, SCOPUS, NHS Evidence and the Cochrane Database of Systematic Reviews (pregnancy and childbirth). Grey literature was identified using PROQUEST (dissertations and thesis search) and Ethos. Searches of reference lists of included studies were manually searched.

### Study selection

The following inclusion criteria were applied during the search: Population specific (i.e. Asian, Indian, Pakistani, Bangladeshi, Kashmiri, Black African, Black Caribbean, Arabian, Traveller [defined as a gypsy or Irish traveller], Chinese, Mediterranean). Maternity Intervention (i.e. maternity based intervention from last menstrual period (LMP) -12 months post-delivery). Site (i.e. U.K., England, Scotland, Northern Ireland, Wales, and Britain); outcomes (e.g. birth weight, intrauterine growth restriction and birth outcome). In addition, papers or reports written in English and published between 2004 and 2014 were included. This publication date range was selected to help ensure that similar national guidance (i.e. National institute for Health and Care Excellence [NICE], [46]) and evidence based clinical practice provides a homogeneous selection of papers, in addition to incorporating a growing awareness of the maternity needs of ethnic minority women in the last decade [45]. The applied exclusion criteria were: Populations other than Asian, Indian, Pakistani, Bangladeshi, Kashmiri, Black African, Black Caribbean, Arabian, Traveller, Chinese, Mediterranean, Interventions that were not LMP – 12 months post-delivery, papers reporting concurrent interventions, region outside of UK (as above), non-measurable outcomes, paper not reported in English, outside 2004–2014, and papers that were literature reviews.

### Data extraction

The full data extraction template was based on Cochrane data extraction [52] and included the following: reviewer name, date, article number, country of origin, publication type, description, methods, design, authors, randomised, method of randomisation, type of intervention, primary aims, secondary aims, concealment, inclusions, exclusions, timing of intervention, duration of intervention and follow up intervals, consider dose (effects and comparability), intervention protocol, control group (specify, population), participant age, compliance, attrition, reasons from attrition, pre-determined outcomes, status in this review (include/exclude/unsure) sample size, setting (inpatient/outpatient/community), outcomes, significance values, statistical methods applied, baseline characteristics of participants, co-morbid factors identified, medical treatments, concurrent risks identified and bias identified. The data extraction template was designed in Microsoft Word by RG and paper copies were manually completed for each study in May 2014. 2 reviewers (RG and NA) undertook the review process, applying an iterative approach to the scoping review question and purpose. Disagreements were resolved through discussion and consensus. The final inclusions were agreed with both reviewers (RG and NA).

### Charting and summarising the findings

In order to systematically chart the data across the heterogeneous studies, a spread sheet was devised, based on the data charting points suggested by Arksey and O’Malley [47] and descriptive-analytical narrative was used to document the findings [49]. In line with the purpose of this review; identifying tailored interventions for BAME pregnant women, the charting process reported the intervention concerned, recorded the participant inclusion criteria, noted whether a comparator was used, recorded whether identifiable confounds were present, documented the studies outcome measures, reported the studies key findings and reported the recommendations from the authors. This process allowed for comparison across the heterogeneous studies.

## Results

**Fig. 1:**
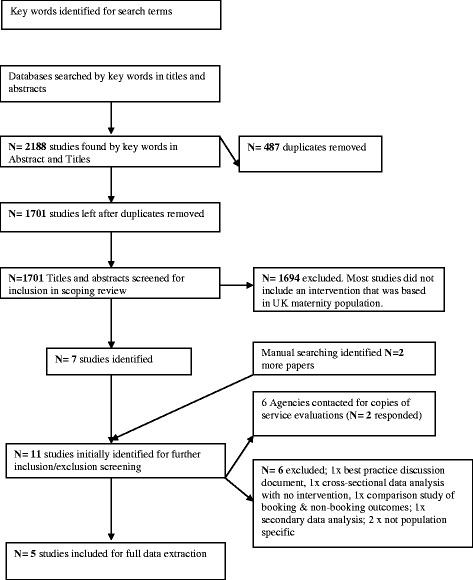
Flow diagram of study review and selection process

After identification of 2188 initial studies, 487 duplicates were removed. One reviewer (RG) undertook initial screening of 1701 identified papers by title and abstract, following the inclusion and exclusion criteria. This resulted in removal of 1694 papers, many of which were either not intervention studies at all, or had not been conducted in the U.K. (the vast majority of the initial identified studies were HIV related and based in the African continent) or were not specific to the maternity population. This left 7 studies. In addition, manual searches identified a further 2 papers and 6 agencies were contact for details of service evaluations (as per the grey literature inclusion criteria). However, only two agencies responded (Haalma, Leeds NHS Trust and Yorkshire and Humber Innovation Education Cluster; Maternal and Infant Health Team). This resulted in 11 identified papers for a more detailed review. The selection process is depicted in Fig. 1.

As suspected, the 11 papers were heterogeneous; the papers ranged from random controlled trails [53–55] to a service report [56]. It transpired that several study populations were heterogeneous; including a broad inclusion criteria and not BAME specific, however, since they were initiated in areas of high BAME residence [53–55] (and the ethnic diversity of the population of these geographical intervention sites were subsequently checked using census data from the Office for National Statistics [57] to ensure that inclusion of papers in this review incorporated interventions that included BAME women). 3 papers were discussed at length by 2 reviewers (NA and RG), whereby it was decided to include these in the final review since the interventions were operated in regions with high ethnic populations (i.e. Bolton, Camden and Islington and ‘inner-city’ with ethnicity reported at 40 %). In addition, their inclusion demonstrates the diversity of maternity interventions offered, as well as showing the paucity of ‘specific’ interventions. Consequently, the final scoping review included 5 papers and excluded 6 papers. The exclusions were as follows: one paper was a best practice discussion document, one paper was a cross-sectional data analysis with no intervention, one paper was a comparison study of booking & non-booking outcomes, without a specific intervention; one paper was a detailed secondary data analysis and two papers were not population specific and failed to detail ethnicity data.

Table 2 shows the final included studies characteristics. The studies ranged from 2004 to 2011. They included participants ranging from *N* = 160–1441. The geographical locations were all urban areas of diverse populations and included, Newham [53], Camden and Islington [55], Bolton [58], West Yorkshire [56] and ‘inner-cities’ not specified [54]. The target populations included; pregnant women of all ethnicities (in an area of high diversity) [53], pregnant women who were less than 19 weeks and 6 days gestation and who did not have a record of their thalassemia status on their medical records [54], mothers who delivered in the six month period of January – September 1999 [55], teenage parents [58] and ‘vulnerable’ and hard to reach women [56]. The employed research designs were sequential mixed methods (cross-sectional and qualitative methods) [53], cluster randomized controlled trial [54] and randomized controlled trial (parallel group with 3 arms) [55]. The remaining two included reviews were service interventions (non-published) [56, 58].

**Table 2:** Included studies characteristics

Authors	Target population	Location	Participants (N)	Method
Austin, 2011 [53]	Pregnant women, all ethnicities included (only 17 % population described as white British in Newham [ONS, 2011])	Newham, East London.	219	Sequential mixed methods (cross-sectional and qualitative).
Dormandy et al., 2010 [54]	Pregnant women <19 weeks, 6 days gestation No record of SCT^a^ status	U.K. – inner cities (not specified) with high percentages of BAME women (at least 40 %)	1441	Cluster Randomised Controlled Trial
Wiggins et al., 2004 [55]	Mothers who delivered between 1st Jan 1999-30th Sept 1999	Camden and Islington, London.	731 (341 = “non-white” and 181 non English speaking participant	Randomised Controlled Trial
Littler, 2010 [58]	Teenage parents	Bolton	160	Multi-Agency intervention^b^
Khan, 2008 [56]	Vulnerable and hard to reach pregnant women	West Yorkshire	286	Service intervention^c^

^a^Sickle Cell Thalassemia (SCT). ^b^ Multi-Agency care pathway for teenage parents. ^c^ Befriending/advocacy service for vulnerable and ‘hard to reach’ women in the ante/post natal period

At the onset of this scoping review, it was determined that an iterative process would be necessary to address the heterogeneous nature of the identified studies [48]. Unexpectedly, the included studies are of either a mixed methodology or quantitative origin, thus facilitating data to be extracted and presented in both a numerical and narrative format. Table 3 charts the studies in more detail, including the results of the individual interventions.

**Table 3:** Charting of the included studies

Author	Intervention	Participant inclusion criteria	Comparison group?	Confounders	Outcome measures	Results	Author recommendations
Austin, 2011 [53]	Dietetic referral for pregnant women with high/low BMI^*^at booking. Personalised dietary and exercise advice from dietetic service every 4–6 weeks throughout pregnancy	Pregnant women with BMI^*^at booking <18.5 or >30	None	Potential confounds: participant descriptive statistics not presented, no information on SES variables, education.	Primary outcomes: Birth outcomes and frequency of dietetic interventions taken	Primary measures: 2 or more interventions with dietetic services showed improved birth outcomes (less infant mortality and no low birth weight). (*n* = 219; 7 adverse outcomes reported[LBW = 4, stillbirth = 3])	Initial results suggest that early dietetic intervention may improve birth outcomes in Newham.
				Individual multiple risk of adverse birth outcome not shown	Secondary outcomes: Qualitative satisfaction questionnaire	Secondary outcomes: 86 % of attendees rated the advice as “very good” or “good”.	More research is required.
Dormandy et al., 2010 [54]	Universal SCT^1^Antenatal Screening in primary care (at first booking) 3 methods tested.	Attendance at participatory surgeries, planned to continue pregnancy, pregnancy gestation <19 weeks and 6 days when first seen in primary care, there was no written record of SCT status and gestational age based on definite LMP date.	3 groups compared for effectiveness, feasibility and acceptability	Potential demand characteristics –only 62 % of health care professionals attended training to deliver the SCT screening invitation intervention, indicating 38 % of staff did not receive training and this may have impacted on the uptake, both positively and negatively.	Primary outcomes: timing of screening, (proportion of women screened before 10 weeks (70 days). Date calculated from LMP^2^	Proportion of women screened within 10 weeks was 2 % (9/441 in standard care, 24 % (161/677) primary care with parallel testing and 28 % (167/590) in primary care.	Research is needed to understand the impact of gestational age on screening uptake and subsequent reproductive decision making.
	Group 1: primary care testing with simultaneous offer of father testing				Secondary outcomes: rates of informed choice and awareness of fathers carrier status at 11 weeks gestation	The number of women screened by 70 days (10 weeks) 3 % (3/90) standard care; 47 % (321/677) in primary care with parallel testing and 48 % (281/590) in primary care using sequential testing.	More research is required to understand why women failed to have SCT screening.
	2. In primary care with offer of follow up father testing if mother a carrier					The percentage of women screened within 26 weeks was reported to be similar in all 3 groups; 73 % standard care; 84 % in primary care with parallel testing and 82 % in primary care with sequential testing	Limited uptake of father testing results in unclear carrier status and reproductive decisions are not considered.
	3. In secondary care with follow up father testing, if mother a carrier.						Other models of screening may facilitate an improved uptake and these need to be explored.
							This study suggests that antenatal screening for SCT is not negatively impacting on emotional wellbeing, however this needs more research
Wiggins et al., 2004 [55]	Levels of Social Support post-natal	Women who gave birth in Camden & Islington between 01/01/99 – 30/09/99.	“usual care” of routine health visitor support (1× home visit) at infant age 10–15 days. Other home visits are only made if a risk is determined; otherwise contact is made at primary care clinic.	HV were recruited and trained for the RCT	Child injury, Maternal smoking status and maternal wellbeing at 12 and 18 months with follow up self-report questionnaire or interviews.	Both intervention groups were demographically well matched.	The SHV intervention was found to be popular and showed some improvement in secondary outcomes. This suggests that increase social support from health visitors may improve maternal and family wellbeing but further research is needed.
	To improve infant and maternal outcomes. 2 arms.	(exclusion if baby died, mother moved away, baby or mother unwell or interpreter unavailable)		Uptake between the 2 groups was imbalanced; 94 % SHV vs 19 % CGS	Secondary outcomes included access to healthcare services and financial services, maternal and child health and the self-reported experiences of feeding and motherhood, assessed using self-report questionnaire or interviews	Response rates were 90 and 82 % at 12 and 18 months respectively.	There is a need to develop and test more culturally specific interventions
	1, SHV^3^12 months of monthly supportive and listening visits from 10 weeks old. SHCV attention on maternal needs			CGS Assignment based on preference, SHV assignment based on geographic proximity to HV base clinic. This may result in bias in the results.		In the SHV group, there was a reduction of GP visits but an increase in use of SHV and social worker services at 12 month follow up.	Research is needed to explore the evidence in delay in subsequent pregnancy found in the study.
	2, CGS^4^ allocation to one of 8 community groups, with drop in sessions, home visits and telephone support for 12 months post- delivery.			A “dose effect” may be evident with increasing contact with support group, regardless of randomisation group		By 18 month follow up, less mothers were pregnant in both SHV and CGS groups compared to “usual care” and SHV mothers were less concerned regards their child’s health.	
				Self- reported measures; there is a risk of under reporting of medical visits and inadequate account of children in receipt of regular medication regimes			
Littler, 2010 [58]	Multi-Agency teenage pregnancy intervention	Not directly specified	Comparisons are made to earlier year’s teenage pregnancy cohorts in the local area and audit data of contraceptive plans.	Possible dose effects from intervention, but frequency and uptake is not reported.	A number of outcomes were reported:	The results were reported:	The intervention was considered successful (through the broad outcome measures) although there is a lack of formal evidence reporting of this intervention, which should attend more closely to the demographic profile of attendees of the service, to ensure that it reaches all sectors of the community, including BAME.
				Contraceptive plans are as intended and not a measure of actual behaviour	C-Section rates, breast feeding uptake, Number of contraceptive plans in place; subsequent pregnancy rates; use of services by teenagers; Referral for social problems; uptake of continued or further education.	C-Section rates decreased (2008 – 2009), Increased breast feeding (33.1–44 %), increased numbers of contraceptive plans in place, reduction in subsequent pregnancy rates (15–8.2 %), increased use of services by teenagers, early referral for social problems 54 % mothers reported to be in education or training following birth of baby.	The redesigning of original services allowed the new service to be developed with no new investment.
							Service users gave positive evaluations.
Khan, 2008 [56]	‘Haamla Service’ befriending, advocacy and support service for vulernable and hard to reach women in West Yorkshire	Not clearly defined;	none	Highly likely – this intervention has no comparison or control group.	Attendance rates in different Haamlaserice sectors (GP surgeries, hospital ward visits, attendance by electoral ward data, ethnicity data (hospital ward, antenatal groups)	Various attendance rates and service activity presented as percentages and frequencies.	A comprehensive service evaluation is required to determine the return on investment in real terms including length of admission, frequencies of admissions and late booking complications leading to adverse outcomes.
		Vulernable (migrants, asylum seekers, refugee’s); Hard to reach (including BAME women).		There is no uniform service and no measurable service outcomes.	% of origins of referrals, gestation period at time of access,	Total women accessed service in 2006 = 286	
				Reliability and validity cannot be established due to lack of methodological rigor.		The majority (66 %) of service involved information giving, several ethnic groups were reported (Pakistani, Bengali, Indian, Black African, Black Caribean, Black other, Chinese, White, Other, Not known) with Pakistani representing the largest number of attendees for hospital ward support in 2006, 2007, 57 % of women seen in GP surgeries were of Pakistani origin, the majority of referrals came from community midwives (32 %) and generated by internal referrals through the hospital (21 %)	

^1^Sickle Cell Thalassemia (SCT). ^2^Multi-Agency care pathway for teenage parents. ^3^Befriending/advocacy service for vulnerable and ‘hard to reach’ women in the ante/post natal period. ^4^Community group support (CGS)

A brief summary of the included results and recommendations follows: the Dietetic service [53] was aimed at pregnant women with high or low BMI at booking (in Newham) found demonstrable reductions in lowering infant mortality rates and no incidences of low birth weight (*N* = 219), although the population sample was small, this does indicate that extra support does have tangible impact on health outcomes, although a more extensive study would need to confirm these early indications (see Austin, 2011). Moreover, this study benefited from using a mixed methods approach resulting in more detailed results than a mono design would typically yield [60, 61]. However, the authors did not detail socioeconomic variables of the participants which may have acted as a mediator to the results; for example, did the women who attended the dietetic service have higher levels of education?

The second included intervention Dormandy and colleagues, [54], tested the timing and location of screening uptake for sickle cell and thalasseamia screening (SCT). Using a cluster randomised control trial, the authors tested 3 methods of screening; in primary care with simultaneous offer of paternal screening; in primary care with offer of subsequent father testing or finally, in secondary care with follow up of father testing, when the mother is identified as a carrier. The authors found a 24 % uptake in primary care with parallel partner testing and 28 % in primary care with sequential partner testing. Further, 48 % of women were tested in primary care using sequential partner testing, compared to 47 % in standard care. The recommendations from this study included the need for further research to understand why women fail to use the screening services and to develop new models of screening delivery to achieve a higher uptake including partners.

Next, Wiggins and colleagues [55] assessed different levels of social support against infant and maternal outcomes, including maternal smoking and wellbeing. Using a Support Health Visitor (SHV) (monthly visits from 10 weeks old to 12 months) compared to community Group Support (CGS) telephone and drop in clinics, offered for 12 months post delivery. Their results showed that in the SHV group, GP visits reduced but social worker referrals increased. Both groups had less mothers in a subsequent pregnancy at 18 months compared to ‘usual care’. Overall, the SHV group was found to be popular and demonstrated improved secondary outcomes (accessing healthcare, financial services, child and mother health, self reported feeding and experiences of motherhood). The authors concluded a requirement to develop culturally competent interventions in addition to further research to understand the delay in subsequent pregnancy.

In Bolton, a Multi-Agency Teenage pregnancy service was developed by redesigning the original service [58]. This intervention is poorly documented and as such, poses a challenge to report. In addition, there are no demographic details of participants and therefore it is unclear exactly how many BAME women accessed this service. However, Bolton is a diverse area and Pakistani and Bangladeshi women are reported to start child bearing during their teenage years and so of including the intervention in the scoping study, it was assumed some service uptake may have been from BAME women, although this fact is unclear [57, 61]. Moreover, the paperwork shows that comparisons are made to earlier year’s teenage pregnancy data, which may suffer from discrepancies in cohort effects and local environmental influences [14, 62]. The Multi-Agency Teenage Pregnancy intervention identified a reduction in caesarean section rates, an increase in breast feeding, an increase in early identification of complex social problems, increased numbers of teenage pregnant mothers with contraception plans in place (prior to delivery). Furthermore, this intervention boasted of 54 % of teenage mothers in education or training, and overall the Multi-Agency Teenage Pregnancy intervention was considered a success.

Finally, the Haamla service was included and reviewed, a befriending and advocacy service aimed at vulnerable and hard to reach women, and as such is the only specific intervention solely addressing the scoping reviews target population of BAME (but did not exclude white participants). This report presented a number of frequencies and percentages detailing service uptake by electoral ward, referral and uptake by clinic, hospital or primary care. In addition, the report showed the majority of service users were Pakistani but other BME groups were also represented (e.g. Bengali, Indian, Black Africa, Black Caribbean, Chinese etc.). The Haamla service report recommended the need for a more extensive service evaluation in addition to the need of increased staff to facilitate the service for the local populous, which has a high BAME population.

## Discussion

The present scoping review has found that there is a paucity of rigorous research and tailored practice interventions addressing specific maternity interventions for BAME women in the U.K. A review of the included studies characteristics (see Table 2), found that the target population (i.e. BAME pregnant women) were included within the broader maternity population of each intervention, by means of inclusion in geographic areas of low numbers of white majority (i.e. Newham) [53] or 46 % inclusion of BAME women evidenced in Camden and Islington, London [55], and in teenage parents in Bolton [58], although only one intervention was aimed solely at the BAME population [56].

Following the methodological approach for scoping reviews from Arksey and O’Malley [47], this review has charted the different participants included and documented the relevant inclusion criteria, methodologies implemented, presence of control or comparison groups, noted potential confounds, documented main outcome measures, recorded main results and highlighted key studies’ recommendations. The included interventions were as expected, found to be heterogeneous. While no patterns or trends are evident within the charted data of identified interventions for BAME pregnant women, the interventions did all report ‘positive outcomes’, consequently, the included interventions were perceived as being beneficial to the target populations, although more rigorous research is needed to determine their efficacy [47, 48].

It is accepted, that there may be interventions in operation at a local level across the U.K., targeting BAME pregnant women, however, since these have not been documented and published, the sharing of best practice has been restricted and these interventions were not identified in the present search. Furthermore, a lack of service evaluation to demonstrate unequivocally the benefit and value of tailored interventions to vulnerable and at risk pregnant women, as seen in the Haamla service (Leeds) and Multi-agency teenage pregnancy service (Bolton) further hinders the progress of developing and justifying specific maternity interventions for high risk BAME women.

The discussion above shows that current maternity interventions are diverse and do include BAME women although services are not always a specifically dedicated to their culturally distinct needs. Only one study was directed to the increased risks of women identifying as BAME origin, by virtue of the prevalence of sickle cell and thalassemia found in Black, Mediterranean and Asian individuals [54]. The Haalma service was directed toward BAME women, but did not address any specific risk factors for adverse maternal or infant outcomes [56]. The other studies included BAME women through the intervention sites (i.e. areas with high BAME populations, such as Newham). One intervention study [53] focussed on high or low BMI pregnant mothers and early dietetic intervention, a known risk factor for co-morbidities such as (gestational) diabetes mellitus, which is associated with increased incidence of stillbirth or congenital abnormalities [44, 45, 63]. The interventions do go some way to addressing the differences evident in risk factors for BAME women experiencing adverse maternal or infant outcomes. Therefore the simple answer to the current question of ‘what special maternity interventions exist for BAME women, at high risk for adverse birth outcomes, in the U.K.?’ is that currently there are a lack of specific interventions designed to support BAME pregnant women during their pregnancies, despite a wealth of evidence showing that certain ethnic groups (e.g. Black African, Black Caribbean and Pakistani) residing in the U.K. are at higher risk of adverse outcomes, such as pre-term delivery (<37 weeks), low birth weight (<2500 g) or co-morbid maternal complications such as pre-eclampsia, diabetes, obesity or hypertension [61, 64–66]. There is a clear need to develop specific culturally relevant maternity services, to meeting the growing needs of BAME pregnant women in the U.K., in order to reduce the persistent health inequalities and improve both maternal and infant outcomes [61, 67, 68].

There are some limitations in the present scoping review. The search was restricted to English language however, as the search was concerned with the UK context. In addition, this review implemented the highly specific PICO criteria and therefore it is possible that some studies have been omitted. Consequently, it is recognised that they may be some valid and useful interventions in operation in the UK which were not identified and therefore are not included in this scoping review (as a consequence of being outside the specificity of PICO, or having not been published or formally written up), and therefore not reflective of the wider clinical picture. Nevertheless, this does confirm that there is a paucity of research, empirical writing and evaluation reports of specialised antenatal services targeting BAME women during pregnancy.

At present, we are a long way from being able to produce a rigorous systematic review of antenatal interventions, aimed at reducing adverse outcomes in BAME women or a robust meta-analysis of the efficacy of a given targeted maternity interventions for BAME expectant women, due to a paucity of published research and service evaluations in the area. Moreover, if this lack of documented evidence of tailored interventions for reducing risk in BAME women is reflective of the wider clinical picture of maternity provision in the U.K., this highlights the need that local maternity services in the U.K. ought to be modified to better accommodate the needs of high risk BAME women, through specific and culturally competent interventions, whilst meeting the needs of the wider population and other vulnerable groups, such as recent migrants or asylum seekers [22, 46, 69].

## Conclusions

BAME women have been shown to be at increased risk for adverse birth outcomes, despite residing in a country providing ‘free’ antenatal care. However, a plethora of research has shown that a number of barriers and facilitators exists that prevent BAME from utilising the mainstream maternity services; consequently there is a need for commissioners to amend current service provision to more culturally competent interventions. The results of the scoping review found only 5 papers that met the inclusion criteria and the papers were heterogeneous in type. The included papers were small-scale and some lacked rigor, but they generally showed favourable outcomes, suggesting that targeted interventions for BAME women may have beneficial outcomes.

This demonstrates that very few BAME women had access to *specific* maternity interventions, even when reviewing a ten year period of retrospective research and clinical evidence as shown by the paucity of papers meeting the inclusion criteria. However, it is likely several local-level initiatives exist, aimed to support BAME pregnant mothers and whose outcomes have not been rigorously tested, reported or published. As a result, this will perpetuate inequalities and hinder the development of culturally competent maternity services. At national policy level, further consideration is required as to how the results of research studies shape future policy and guidance to commissioners to ensure that culturally competent service provision is seen as integral to effective service commissioning rather an ‘add-on’.

## Footnotes

1 For the purposes of this paper ethnic (and ethnicity) identity is defined as encompassing cultural, religious, language common ancestry, shared territories and physical appearance [71–73]

2 In the UK the ethnicity of infants is identified by mother’s ethnicity and is recorded in the Birth Notification Application system [13, 74]
